# Hand Motion Detection in fNIRS Neuroimaging Data

**DOI:** 10.3390/healthcare5020020

**Published:** 2017-04-15

**Authors:** Mohammadreza Abtahi, Amir Mohammad Amiri, Dennis Byrd, Kunal Mankodiya

**Affiliations:** 1 Department of Electrical, Computer and Biomedical Engineering, University of Rhode Island, Kingston, RI 02881, USA; amir.amiri@temple.edu; 2 Department of Physical Therapy, College of Public Health, Temple University, Philadelphia, PA 19140, USA; 3 Interdisciplinary Neuroscience Program, University of Rhode Island, Kingston, RI 02881, USA; byrd.dennis@gmail.com

**Keywords:** motion detection, fNIRS, SVM, hand motion

## Abstract

As the number of people diagnosed with movement disorders is increasing, it becomes vital to design techniques that allow the better understanding of human brain in naturalistic settings. There are many brain imaging methods such as fMRI, SPECT, and MEG that provide the functional information of the brain. However, these techniques have some limitations including immobility, cost, and motion artifacts. One of the most emerging portable brain scanners available today is functional near-infrared spectroscopy (fNIRS). In this study, we have conducted fNIRS neuroimaging of seven healthy subjects while they were performing wrist tasks such as flipping their hand with the periods of rest (no movement). Different models of support vector machine is applied to these fNIRS neuroimaging data and the results show that we could classify the action and rest periods with the accuracy of over 80% for the fNIRS data of individual participants. Our results are promising and suggest that the presented classification method for fNIRS could further be applied to real-time applications such as brain computer interfacing (BCI), and into the future steps of this research to record brain activity from fNIRS and EEG, and fuse them with the body motion sensors to correlate the activities.

## 1. Introduction

Brain is the most complex organ in the human body, consisting of millions of neurons and cells. Our brain controls almost every single task we do. The brain processes our senses of what we hear, see, smell, and our feelings. Also, the time we move and make any movements in the body, from a single hand movement to dancing, it is the result of our brain receiving, processing and sending messages to and from different organs [1].

Movement disorders are the result of any damage or malfunction in the nervous system or muscles which need complex interaction between each other for any kind of movements [2]. The incidence of the movement disorders, which refers to the number of new cases at each year who diagnosed with movement disorders [3], is increasing and it is projected that there would be an extreme increase between the years 2010 and 2050 [4]. Various methods are available for diagnosing or treatment of the movement disorders, and brain monitoring can be one of the good choices among them.

There are many different types of non-invasive brain monitoring techniques such as positron emission tomography (PET), single-positron emission computed tomography (SPECT), and functional magnetic resonance imaging (fMRI) which their main drawback would be their immobility. On the other hand, there are some other techniques such as magnetoencephalography (MEG) and electroencephalography (EEG) that are portable and measure the cortical electromagnetic and electrical activities respectively, directly by placing the electrodes on the scalp surface [5]. The main drawback of these recent methods is the motion artifact contaminated data while doing some movement experiments. One of the portable non-invasive brain monitoring methods is functional near infrared spectroscopy (fNIRS) which detects the hemodynamic changes in cortical regions of the brain based on the optical principles [6,7]. Motor tasks, in particular, have been a subject of fNIR studies because the technique is well-suited for examining the associated cortical areas, the Pre Motor Cortex (PMC) and Primary Motor Cortex (M1).

In this study, we have recorded data with fNIRS from the motor cortex region of healthy subjects, while they were asked to perform movement tasks that are borrowed from Parkinson’s disease screening protocol along with some rest periods, during which the participants need to be stationery. We then applied a lowpass filter to reduce environment noise from the data and then underwent through numerous classification methods to detect the rest and action periods.

## 2. Background and Related Works

fNIRS competes with other imaging techniques such as fMRI, EEG, and PET. An advantage of fNIRS imaging in the case of the PMC and M1, is that those areas are on the outer cortices, which is within the blood oxygenation level depravation (BOLD) scanning range for fNIRS [8]. While a fMRI examines BOLD with a magnetic field, an fNIR emitter shines infrared light which is in the range of 700–900 nm from outside, on the surface of the scalp, through the skull, meninges, and cortex. This light refracts as it interacts with Oxygenated Hemoglobin (HbO2) and Deoxygenated Hemoglobin (Hb). The detector components receive the refracted light and are able to distinguish the relative concentrations of each [9].

**Figure 1:**
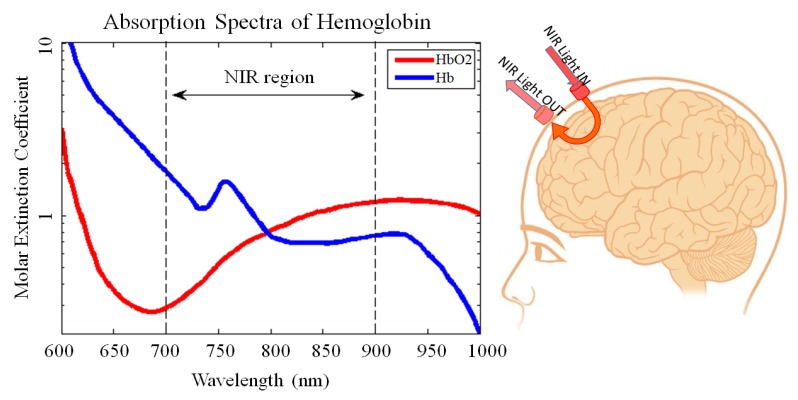
A schematic of how the fNIRS works with the absorption spectra of HbO2 and Hb.

Oxygenated Hemoglobin (HbO2) and Deoxygenated Hemoglobin (Hb) are almost the strongest absorbers of light at the near infrared (NIR) spectrum, while all the layers on the head including the skin, bone, tissues and lipid are transparent. fNIRS takes advantage of this phenomenon and uses NIR light to monitor the blood flow in the cortical regions of the brain and provide hemodynamic responses of the brain based on the absorption of HbO2 and Hb [10]. Figure 1 illustrates a schematic of how the fNIRS system records the hemodynamic response by sending NIR light into the cortex and receive the reflected attenuated light through the detectors along with the absorption spectra of HbO2 and Hb.

A number of studies have used fNIRS to monitor changes of cerebral oxygenation as a response to different tasks such as visual [11,12], cognitive [13,14,15,16] and motor [9,17,18,19,20,21] in the recent twenty years.

Some studies have been performed with the goal of movement classification based on the fNIRS signal and providing brain computer interfaces for detecting different movements and improving the prediction accuracy of the system in small practice settings.

- Naseer and Hong et al. [22], acquired signal from the right and left primary motor cortices of ten healthy participants by using a continuous wave fNIRS system, with the goal to discriminate the right and left wrist flexion imageries. They have used linear discriminant analysis classifier and achieved the average classification accuracies of 73.35% and 83.0% for the right and left wrist motor imageries, respectively, during the 10 s task period. Also, by confining the analysis time to 2–7 s span within the overall 10 s task period, the average classification accuracies were improved to 77.56% and 87.28% respectively.
- In a report by Sitaram et al. [23], the use of a continuous wave 20-channel NIRS system over the motor cortex of 5 healthy volunteers was reported in order to measure oxygenated and deoxygenated hemoglobin changes during left-hand and right-hand motor imagery. It is mentioned in the report that support vector machines has been used to classify left-hand imagery from right-hand imagery with an average accuracy of 73% for the volunteers.
- Fazli et al. [24] applied both fNIRS and EEG methods simultaneously in a real time Sensory Motor Rhythm (SMR)-based BCI paradigm, involving executed movements as well as motor imagery and show that simultaneous measurements of NIRS and EEG can improve the classification accuracy of motor imagery in around 90% of the subjects and an average of 5% increase in the performance.
- In a report by Almajidy et al. [25], fNIRS, EEG and tripolar concentric ring electrode electroencephalography (tEEG) has been used to control a 2-D BCI and different features extracted from the signals. Linear Discriminant Analysis (LDA) has been used to classify different combinations of the features and 85% accuracy has achieved.
- In another report by Power et al. [26], classification of prefrontal activity due to two cognitive tasks, specifically mental arithmetic and music imagery, based on the fNIRS data is reported. It is mentioned that an average accuracy of 77.2%±7.0 across the participants is achieved to classify mental arithmetic and music imagery.

## 3. Data Acquisition

### 3.1. Participants

In this study, we have recruited seven healthy participants all aged between 20 to 33 years old. All the participants were recruited from the University of Rhode Island. They signed consent forms based on the institutional review board (IRB) requirements. A brief explanation of the subjects can be found in Table 1.

### 3.2. Protocol

Participants were asked to do several motion tasks including flipping the right hand, rotating the right hand from the wrist, drawing circles on a paper and walking with some rest periods in between of each task, which would be helpful in analysis of motion for patients suffering from Parkinson’s disease. In this study, we specifically focused on just one task involving the activity of flipping the right hand. The duration of the tasks are 30 s followed after 30 s of rest, which is staying stationery. For participants 1 to 4, they have completed this task for 15 trials with the duration of 30 s, and a rest window time of 30 s in between of each trial. Participants 5 to 7, could finish only two trails, therefore we did not add the data from these participants in the processing part in this study.

**Figure 2:**
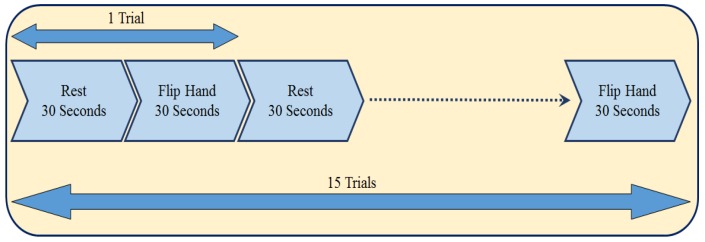
An illustration of the timings of the trials in the experiment.

The time of a rest period followed by the task period lasts 1 min, and repeating it for 15 times, provides a 15 min data of motion windowed in 30-s intervals of rest and flipping the hand following after each other. Figure 2 shows a time-line of the protocol.

### 3.3. fNIRS Neuroimaging Data

fNIRS data is recorded from the NIRScout System (NIRx Inc., New York, NY, USA), using an 8×8 sensor array on the motor cortex area on the head. We placed the optodes precisely on the motor cortex related areas on the 10–20 montage that yielded 20 fNIRS channels (10 channels in each hemisphere).

**Figure 3:**
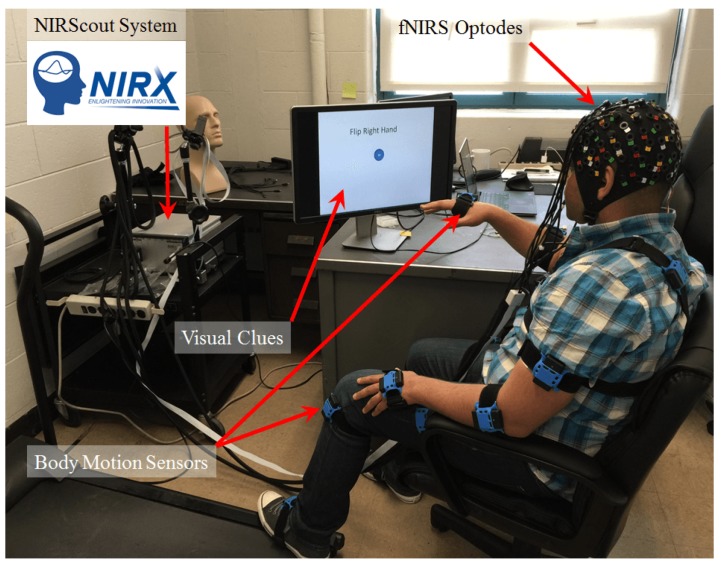
Experiment setup showing the sensors that has been used.

Along with the fNIRS data, we have recorded motion data from Mocap body motion sensors (YEI Technology, Portsmouth, OH, USA). The body motion sensors are wearable and completely wireless. Each sensor records motion data and transfers it wirelessly to the dongles that are connected to the same computer that fNIRS is recording data on. We used 16 body motion sensors which can record the movements from all the body. In this paper, we used the data from the motion sensors for synchronization of the fNIRS data. Figure 3 shows how the setup of the experiment would look like when the participant is wearing the sensors and doing the experiment based on the visual clues on the screen.

## 4. Methods

In this section, the approach and the methods of classification used to classify the rest and action periods from the fNIRS data is described. It is important to mention that the main spectrum of the fNIRS data occurs in lower frequencies less than 1.5 Hz. Therefore, prior to applying any of the methods mentioned here, we applied a 4th order low-pass Butterworth filter with the cutoff frequency of 1.5 Hz in order to remove the environmental noise. Also, regarding the physiological noises such as heartbeat and Mayor waves, we applied a 4th order band-pass Butterworth filter with the cutoff frequencies of 0.1 and 0.5 Hz as suggested in [27,28].

### 4.1. Segmentation

**Figure 4:**
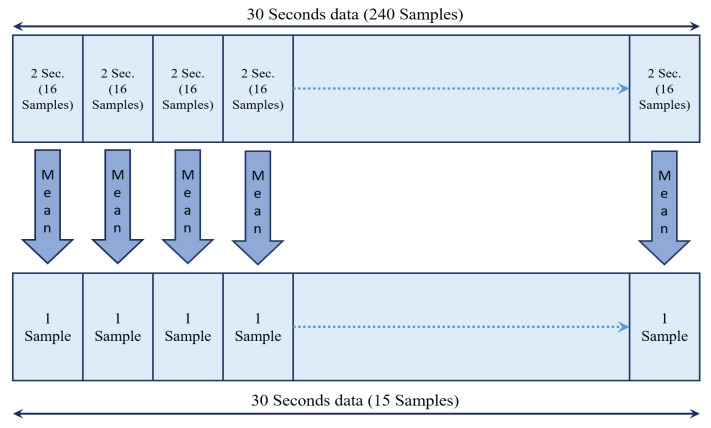
Schematic of the approach of segmentation.

Segmentation and alignment algorithms serve as important preprocessing steps before fNIRS data are applied to the classifier. Therefore, we first use the segmentation and will apply the classifiers to the dataset in order to classify the rest and action periods. In order to explain the approach of the segmentation we used, let us assume we are looking on just a 30-s window of the dataset which is related to either rest or action task. We divided this window of the data into 15 segments, each containing the data for the duration of 2 s, and the segments have no overlap with each other. We then took the mean of all the samples of the data in each of the 2-second segments. Each 2-second segment, contains 16 samples of the fNIRS data. Therefore, we take the mean of these 16 samples and keep the mean as our new data point. Figure 4 shows how the segmentation has been done.

By applying the segmentation, we reduced the size of the dataset, and for each 30-second window of the data, we had only 15 data points instead of all the 240 samples of the original data. We then applied the different classification methods on the reduced size segmented dataset and got the accuracy of the system for classifying the rest and action tasks.

### 4.2. Classification

Support Vector Machine (SVM) is a supervised learning model which uses a nonlinear mapping to transform the original training data into a higher dimension. It searches for the linear optimal separating hyperplane in this new dimension (that is, a “decision boundary” separating the tuples of one class from another). With an appropriate nonlinear mapping to a sufficiently high dimension, data from two classes can always be separated by a hyperplane [29].

The general concept of the SVMs is that the system trains itself based on a training dataset which can be a part of the original dataset that is labeled into two different categories. Then, after the system is trained, it will be tested on the other part of the original dataset to predict the labels of the data, and by comparing the predicted labels and the original labels, we can find the accuracy of the system. In this study, we used several learning algorithms to be implemented in the support vector machines. By applying the Lagrangian optimization theory to a linear support vector machine, and using the Kernel functions, we could classify the datasets which are not linearly separable, while the nonlinear support vector machines retain the efficiency of finding linear decision surfaces, but allow us to apply them to not linearly separable datasets. It is also possible to change the margins of the classifiers and change the complexity and accuracy of the systems. In general, large margins make the system less complex but on the other hand will let the system to make more errors, resulting to less accuracy. This can be achieved by changing a variable called Cost constant in the classifier models.

Table 2 shows different learning algorithms that have been used in this study with their complexity index which we will refer to them later in the results section.

In this work, we used the 10 channels of the fNIRS data related to the left hemisphere of the brain. The reason is that the experiment is based on the right hand movement which is believed to activate the motor cortex area in the left hemisphere. Thus, we focused on the channels related to this hemisphere. Data has been labeled based on the rest and action tasks, with the label 0 for the rest periods and label 1 for the action periods. In other words, for each 30-second window of the data which contains 240 samples, we have labeled the data 0 or 1 for the rest and action tasks, respectively.

### 4.3. Evaluation

Recalling from the explanation of the support vector machine, it needs to cut the original dataset into two parts, one for training itself and one for testing and providing the accuracy, which is called evaluation. On the evaluation aspect of the classification, we used different methods of training and testing the system. The methods that we have used are Hold-Out and *k*-Fold Cross-Validation. Let us spend a few words explaining the differences between these methods. The Hold-Out method simply takes one portion of the dataset for the training and holds the other portion for testing the accuracy. The ratio of these portions can be defined by the user and we defined the portions to be 2/3 and 1/3 for training and testing, respectively. Therefore, the system takes two-third of the dataset and trains itself, and then predicts the labels of the other one-third portion of the dataset and at the end, compares the predicted labels with the original labels and provides the accuracy. In *k*-Fold Cross-Validation, the system divides the dataset into *k* different folds which the length of each fold is the same as the others and no folds have overlap with each other. The concept is that the system uses each of these folds for testing while getting trained from the other folds, and at the end, provides the accuracy of the system which is the average of all the accuracies on different folds.

In this paper, we present various learning algorithms with different types of evaluations on the full datasets from the first four participants. We could not test classifiers on the datasets of participants 5 to 7, because they could only perform two trails. Classifying the data with low number of observations is not feasible. As it is explained in the previous section, the duration of the experiment is 15 min which is divided into 15 trials that contains 30 s rest period followed by 30 s action period. The sampling rate of the NIRScout system is 7.81 Hz, which provides 7029 samples for the whole duration of the experiment. Applying different classifiers to the datasets, found the best model with highest accuracy.

## 5. Experimental Results

**Figure 5:**
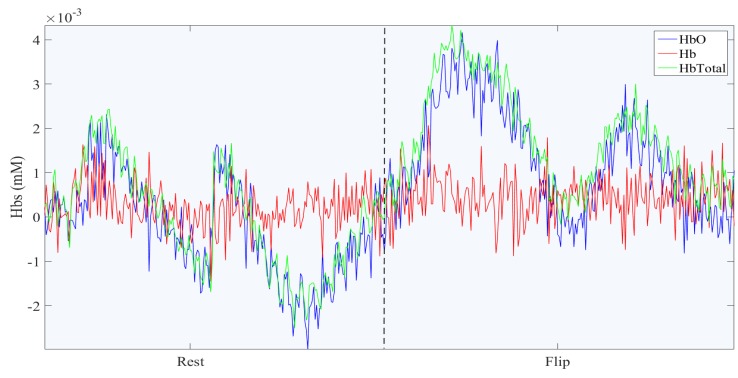
HbO, Hb and HbTotal of a trial of rest followed by flipping the right hand, related to one channel of fNIRS data located on the motor cortex of the left hemisphere.

This section reports experimental results and discusses the procedure of classifying the fNIRS data recorded from the motor cortex while the participants were flipping the right hand. Figure 5 shows the Oxygenated Hemoglobin (HbO), Deoxygenated Hemoglobin (Hb) and Total Hemoglobin of one fNIRS channel in a trial of rest and flipping hand action. As it can be seen, the HbO increases when the flipping hand action starts, which also leads to the increase in total Hemoglobin.

The first set of our observations, involves the grid search in order to find the best model of classification for this kind of data. To achieve this goal, we applied 12 models of classification mentioned in Table 2 with different types of evaluation methods mentioned earlier. We applied these methods on the full-length filtered dataset as well as the segmented reduced size dataset of the first four participants. Regarding the evaluation of the systems, we used Hold-Out method with the portion of 2/3 and *k*-Fold Cross-Validation with *k* equal to 10 for the full-length dataset and the segmented dataset.

**Figure 6:**
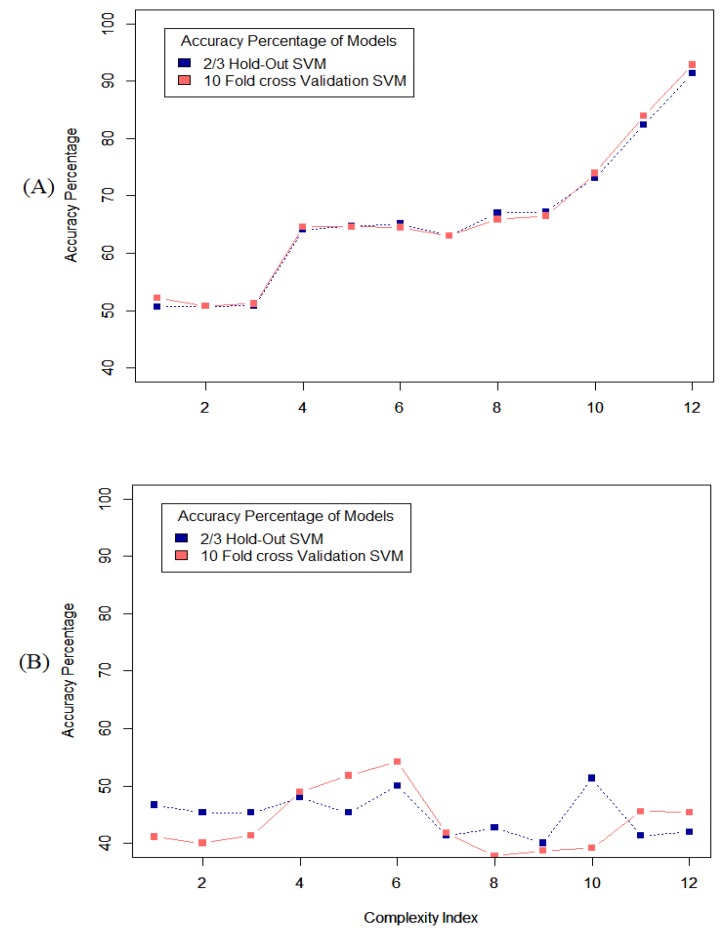
Accuracy of different methods of classification applied on (**A**) full length dataset; (**B**) segmented dataset, on the data recorded from one of the first four participants. Refer to Table 2 for complexity index.

The results of the grid search on the datasets from each of the first four participants show that the classification methods work better on the full-length datasets compared to the segmented datasets. Although segmentation can be counted as a good tool to implement classification methods in many different aspects, but in this case and this type of data, it has some drawbacks. Figure 6, shows the accuracy of the system using different types of classification methods applied to the full-length dataset and segmented dataset from one participant and reveals that the performance of the system is significantly better when applied to the full-length dataset.

Investigating the performance of the system on the data from other participants showed that applying the classification methods on the full-length dataset, produced better results than applying on the segmented datasets. Table 3 shows the performance of different methods on the full-length data for each participant. It can be seen that using the Radial Kernel function with the cost constant of 100, has better performance compared to other classification methods and results in more than 80% accuracy with both the *k*-Fold Cross-Validation, and Hold-Out methods of evaluating the system.

## 6. Conclusions

In this study, we used fNIRS neuroimaging data to classify one action of flipping the hand and the rest time. The data has been recorded from seven participants with 15 trials for four of the participants and 2 trials for the other three. We applied numerous methods of classification on the full-length data as well as the 2-second segmented data on the data recorded from the first four participants who could finish the experiment completely. The results of the grid search, revealed that applying the classification methods on the full-length dataset could provide better accuracy of more than 80%, compared to the segmented dataset. The grid search nominated the SVM with Radial kernel and cost constant of 100 as the best model with the highest performance on the dataset. Both evaluation methods of Hold-Out and *k*-Fold Cross-Validation result in almost the same accuracies, but 10-Fold Cross-Validation has a slightly better performance.

Our conclusion in this paper, indicates that it is possible to distinguish the rest and action periods from the fNIRS neuroimaging data. We believe that our results on the classification are promising and would help further the research of fNIRS in the applications including brain computer interfacing.

This research is the first step going forward to the main goal of the research to fuse the data from the brain and body. The future works involve making the hybrid system of the fNIRS and EEG recording to achieve better resolution, and fusing the data from motion sensors to correlate the muscular activities in parallel.

## Figures and Tables

**Table 1:** Information about participants of the experiment.

Participant	1	2	3	4	5	6	7
Gender	M	M	M	M	F	F	M
Dominant Hand	Right	Left	Right	Right	Right	Right	Right
Hair Color	Black	Brown	Blond	Brown	Brown	Blond	Brown
Hair Length	2 in.	2.5 in.	3 in.	3 in.	12 in.	12 in.	1 in.
Skin Tone	Dark	Light	Light	Dark	Light	Light	Light
Age	33	21	22	22	23	22	21

**Table 2:** Different methods of classification used in this study.

Index	Kernel Name	Kernel Function	Degree	Cost Constant
1	Linear Kernel	$k\left(\vec{x},\vec{y}\right)=\vec{x}\cdot \vec{y}$	–	1
2	Linear Kernel	$k\left(\vec{x},\vec{y}\right)=\vec{x}\cdot \vec{y}$	–	10
3	Linear Kernel	$k\left(\vec{x},\vec{y}\right)=\vec{x}\cdot \vec{y}$	–	100
4	Polynomial Kernel	$k\left(\vec{x},\vec{y}\right)={\left(\vec{x}\cdot \vec{y}+c\right)}^{d}$	2	1
5	Polynomial Kernel	$k\left(\vec{x},\vec{y}\right)={\left(\vec{x}\cdot \vec{y}+c\right)}^{d}$	2	10
6	Polynomial Kernel	$k\left(\vec{x},\vec{y}\right)={\left(\vec{x}\cdot \vec{y}+c\right)}^{d}$	2	100
7	Polynomial kernel	$k\left(\vec{x},\vec{y}\right)={\left(\vec{x}\cdot \vec{y}+c\right)}^{d}$	3	1
8	Polynomial Kernel	$k\left(\vec{x},\vec{y}\right)={\left(\vec{x}\cdot \vec{y}+c\right)}^{d}$	3	10
9	Polynomial kernel	$k\left(\vec{x},\vec{y}\right)={\left(\vec{x}\cdot \vec{y}+c\right)}^{d}$	3	100
10	Radial kernel	$k\left(\vec{x},\vec{y}\right)=e^{-(|\vec{x}-\vec{y}{|}^{2}/2\sigma^{2})}$	–	1
11	Radial kernel	$k\left(\vec{x},\vec{y}\right)=e^{-(|\vec{x}x-\vec{y}{|}^{2}/2\sigma^{2})}$	–	10
12	Radial Kernel	$k\left(\vec{x},\vec{y}\right)=e^{-(|\vec{x}-\vec{y}{|}^{2}/2\sigma^{2})}$	–	100

**Table 3:** The performance of different methods on the full-length data from each of the first four participants.

SVM Method	Evaluation	P1	P2	P3	P4
Linear kernel	10 Fold	49.88%	49.87%	52.16%	52.33%
C = 1	Hold-Out	49.95%	50.64%	50.68%	53.92%
Linear kernel	10 Fold	49.91%	49.90%	50.80%	52.59%
C = 10	Hold-Out	49.87%	50.64%	50.73%	53.84%
Linear kernel	10 Fold	49.13%	49.47%	51.17%	52.19%
C = 100	Hold-Out	49.91%	50.68%	50.81%	53.88%
Polynomial degree 2 kernel	10 Fold	58.88%	63.33%	64.57%	61.58%
C = 1	Hold-Out	59.17%	63.14%	64.12%	61.77%
Polynomial degree 2 kernel	10 Fold	61.28%	62.83%	64.59%	63.24%
C = 10	Hold-Out	60.92%	62.97%	64.67%	62.88%
Polynomial degree 2 kernel	10 Fold	61.68%	63.04%	64.48%	62.93%
C = 100	Hold-Out	61.82%	62.67%	65.10%	62.88%
Polynomial degree 3 kernel	10 Fold	58.95%	63.10%	63.00%	59.70%
C = 1	Hold-Out	57.94%	60.32%	62.97%	59.81%
Polynomial degree 3 kernel	10 Fold	62.28%	67.72%	65.89%	65.50%
C = 10	Hold-Out	61.05%	66.17%	67.02%	65.19%
Polynomial degree 3 kernel	10 Fold	65.93%	69.27%	66.51%	65.55%
C = 100	Hold-Out	63.11%	68.85%	67.23%	64.72%
Radial kernel	10 Fold	66.33%	70.88%	73.93%	69.23%
C = 1	Hold-Out	63.10%	68.61%	73.12%	68.34%
Radial kernel	10 Fold	72.80%	82.33%	83.90%	77.21%
C = 10	Hold-Out	71.42%	79.73%	82.42%	75.64%
Radial kernel	10 Fold	78.98%	90.41%	92.84%	84.15%
C = 100	Hold-Out	76.66%	88.48%	91.30%	82.68%
